# Eosinophilic granulomatosis with polyangiitis (Churg-Strauss syndrome) presenting as diffuse myositis

**DOI:** 10.1186/1471-2474-15-388

**Published:** 2014-11-21

**Authors:** Marc-Etienne Parent, Sandrine Larue, Benjamin Ellezam

**Affiliations:** Department of Internal Medicine, Université de Sherbrooke, 3001 12e Avenue, Fleurimont, QC J1H 5N4 Canada; Department of Neurology, Université de Sherbrooke, 4896 Boulevard Taschereau, Bureau 250, Greenfield Park, J4V 2J2 Canada; Department of Neurology, Notre-Dame Hospital, Université de Montréal, 1560 rue Sherbrooke Est, Montréal, QC H2L 4M1 Canada; Department of Pathology, Sainte-Justine Hospital, Université de Montréal, 3175 Côte-Ste-Catherine, Suite 5938, Montréal, QC H3T 1C5 Canada

**Keywords:** Vasculitis, Myositis, Eosinophilia, Churg-Strauss, Myalgia, Muscle biopsy

## Abstract

**Background:**

Eosinophilic granulomatosis with polyangiitis is a complex multisystemic syndrome with heterogeneous presentation. Most often, there is a clinical history of asthma or other atopic conditions, and current presentation generally includes signs of cutaneous or pulmonary involvement. Very few reports described myalgia or weakness as the chief complaint. Of these, only a few included muscle biopsy evaluation and none showed convincing evidence of primary myositis. We believe this report is the first to demonstrate true myositis in the setting of early eosinophilic granulomatosis with polyangiitis.

**Case presentation:**

This report describes a 74 year old Caucasian man, with no known allergies, presenting severe myalgia, muscle weakness, jaw claudication, and fever. Blood work showed marked eosinophilia and high creatine kinase levels. Biceps brachialis muscle biopsy revealed eosinophilic necrotizing vasculitis and true myositis with myophagocytosis of non-necrotic fibers and strong sarcolemmal MHC-1 overexpression by immunohistochemistry. This patient was successfully treated with prednisone and azathioprine.

**Conclusion:**

Our finding of true myositis in a case of eosinophilic granulomatosis with polyangiitis suggests that primary auto-immunity against muscle fibers, distinct from the secondary effects of vasculitis, can occur in this entity and may represent an overlap syndrome. Early recognition of eosinophilic granulomatosis with polyangiitis in patients presenting with myositis may provide an opportunity to treat the vasculitis before onset of severe multisystemic disease. We recommend the use of muscle biopsy with immunohistochemistry for MHC-1 to confirm the diagnosis of myositis in the setting of eosinophilic granulomatosis with polyangiitis.

**Electronic supplementary material:**

The online version of this article (doi:10.1186/1471-2474-15-388) contains supplementary material, which is available to authorized users.

## Background

In 1951, Churg and Strauss [1] described a syndrome with a relatively uniform clinical picture of severe asthma, fever, peripheral eosinophilia, and symptoms of “vascular embarrassment” in 13 patients initially diagnosed as having polyarteritis nodosa. Churg-Strauss syndrome (CSS) or Eosinophilic Granulomatosis with Polyangiitis (EGPA) is an uncommon form of antineutrophil cytoplasmic antibody-associated necrotizing vasculitis affecting the small-to-medium sized blood vessels. Disease typically follows three phases. The prodromal phase presents as adult-onset asthma, atopic disease, and allergic rhinosinusitis. It precedes the vasculitic phase by 3 to 9 years but intervals as long as 30 years have also been described [2]. Next, is the eosinophilic phase which is usually a subclinical eosinophilic tissue infiltration without necrosis but can sometimes manifest as pulmonary infiltrates or nodules with or without pneumonitis, or as gastroenteritis [3, 4]. The third and final phase is the vasculitic phase, where patients are usually diagnosed. The mean age at presentation varies from 38 to 52 years of age, but can range from 7 to 74 [2, 5]. In a recent large systematic retrospective study of 383 patients with EGPA [5], the most common manifestations at the time of diagnosis were asthma (91.1%), weight loss (49.3%), mononeuritis multiplex (46%), sinusitis or polyposis (41.8%), skin lesions (39.7%), and lung infiltrates (38.6%). In another study and follow-up of 96 patients [3], 94 had asthma at presentation and the remaining 2 developed asthma within the next 12 months of follow-up. A history of allergy is less common when compared to asthma, being described in only 27.2% of patients at presentation [3].

Other than eosinophilia, laboratory data is usually nonspecific and of little help in establishing the diagnosis of EGPA. Eosinophilia usually appears in the second phase of disease, but can also initially present in the prodromal or vasculitic phases [6]. Reported mean levels are 7–8 ×10^9^/L [3, 4]. Interestingly in these studies, some patients had blood eosinophil counts of <0.5 ×10^9^/L at the time of diagnosis, but all were already taking oral CS for asthma control. Similarly, in the Guillevin et al. [3] follow-up, 10 patients had a blood eosinophilia count <1.5 ×10^9^/L, but all were >0.5 ×10^9^/L. Eight of these patients were also already on oral CS. The presence of ANCA is documented in approximately 40% of EGPA [2–5, 7] and appears to be associated with renal and upper respiratory tract involvement, alveolar hemorrhage, purpura, biopsy-proven vasculitis and peripheral neuropathy. The absence of ANCA is associated with pulmonary disease, cardiomyopathy, and fever [2, 3, 8]. The origin of this dichotomy is uncertain but it is hypothesized that the tissue damage in ANCA-negative EGPA is directly related to the presence of tissue eosinophils, whereas the ANCA-positive patients are thought to have endothelial damage in the typical ANCA-associated vasculitis fashion [2].

In 1990, the American College of Rheumatology established the classification criteria for EGPA with a sensitivity of 85% and specificity of 99.7% [9]. It is important to keep in mind that these criteria were established to differentiate patients with EGPA from patients with other forms of vasculitis. Hence, their use in absence of biopsy-proven vasculitis is of little help. Fulfillment of 4 or more criteria is needed for classification as EGPA. The criteria are (1) asthma, (2) eosinophilia greater than 10% on differential white blood cell count, (3) mononeuropathy or polyneuropathy, (4) migratory or transient pulmonary infiltrates detected radiographically, (5) paranasal sinus abnormality, and (6) biopsy with perivascular eosinophils.

Myalgia can be a common symptom of EGPA and has been observed in up to 37-57% of cases of EGPA [2, 3, 5]. However, it is rarely seen as the main presenting symptom and is more commonly observed in the prodromal phase of disease [2, 8] and not typically associated with weakness [3]. It is sometimes accompanied by migratory polyarthralgia although true arthritis is uncommon. In one study, myalgia did not correlate with ANCA status [5]. Unfortunately, CK levels have not been reported in EGPA case series.

Very few EGPA cases of patients having myalgia and weakness as the main presenting complaints have been reported to date. Muscle biopsy descriptions are limited and reveal only vasculitis rather than usual features of primary inflammatory myopathy that would be expected in a true “myositis”, such as phagocytosis of non-necrotic fibers, dense endomysial or perimysial lymphocytic infiltrates, or sarcolemmal overexpression of MHC-1. One patient was a 68 year old woman with a long history of sinusitis and more recent onset asthma presenting with myalgia, mild leg weakness, and elevated CK following strenuous exercise [10]. However, muscle biopsy only showed vasculitis and eosinophilic infiltration but no further inflammatory myopathic features. Another was an 81 year old man with late onset asthma, presenting with a 3-week history of myalgia and weakness with mild CK elevation, reduced nerve conduction amplitudes, and normal EMG [11]. Muscle biopsy showed necrotizing vasculitis with eosinophils in an epimysial medium-sized artery but no endomysial inflammation. Angulated atrophic fibers were present, a histologic sign of denervation. A third report described a 58 year old woman, again with late onset asthma, who presented with a 5-month history of progressive exercise-induced muscle weakness, stiffness and pain, and evidence of axonal neuropathy on electrophysiologic studies. Muscle biopsy revealed necrotizing vasculitis of medium-sized arteries and an extra-vascular granuloma, both associated with eosinophils. Some focal interstitial infiltration with eosinophils was present but no features of inflammatory myopathy were reported [12]. In a retrospective single center study of 19 patients with EGPA, 10 patients were documented as having myalgia but only one underwent biopsy, reported as showing “myositis” with no further histologic description or immunohistochemical analysis to support it [13].

## Case presentation

A 74 year old Caucasian man presented to the emergency room complaining of severe diffuse myalgia, motor fatigability with jaw claudication, a 7-day fever, and a single episode of hemoptysis preceded by increasingly frequent cold-induced paroxysmal dyspnea. Despite being a smoker, he reported only two previous episodes of severe dyspnea with audible wheezing and mild productive cough in the two years prior, both treated with bronchodilators or a short course of CS plus antibiotics. Spirometry demonstrated mild airway obstruction with partial reversibility. There were no drug use or known allergies but he reported a 20-year history of recurrent rhinorrhea and chronic nasal obstruction. History was also remarkable for consumption of bear meat during recent hunting trips within Canada and the United States, but negative for cardiopulmonary, cutaneous, or neurological symptoms such as paresthesia or sensory deficits.

On examination, blood pressure was 117/72 mmHg, heart rate 103 beats/minute, and oral temperature 38.0°C. There was a 2/6 systolic murmur over mitral and aortic areas with radiation to both carotids and the left axilla. Neurological examination showed 3/5 weakness in psoas muscles, 4/5 weakness in upper and lower limbs, and diffuse bilateral myalgia. Sensitivity, tone and reflexes were normal. Examination was otherwise unremarkable.

Laboratory values revealed mild leukocytosis, 18.5 ×10^9^/L (N, 4.5-11.3 ×10^9^/L); marked eosinophilia, 5.07 ×10^9^/L (N, <0.80 ×10^9^/L); hyperCKemia, 3708 U/L (N 15–195 U/L); increased C-reactive protein, 173.7 mg/L (N, <5.0 mg/L); increased rheumatoid factor, 77 kU/L (N, <14 kU/L); increased alkaline phosphatase, 235 U/L (N, 30–105 U/L), and slightly increased alanine transaminase, 86 U/L (normal, 5–55 U/L); Complement C3 and C4 were normal. Myeloperoxidase-specific antineutrophil cytoplasmic antibody (MPO-ANCA), antinuclear antigen (ANA), and cytoplasmic-staining antineutrophil cytoplasmic antibody (C-ANCA) were negative. Serum protein electrophoresis showed a non-specific reactive pattern. Blood, stool, and urine cultures including testing for parasites were negative. Serology for trichinellosis, HIV, HBV, HCV, and CMV were negative, as was EBV monotest. PPD skin test was non-reactive.

Chest x-ray, abdominal ultrasound, and electrocardiogram were normal. Transthoracic cardiac ultrasound showed mild aortic stenosis, mild mitral stenosis, and mild to moderate mitral regurgitation. There was no evidence of endocarditis. Head CT scan showed sinusitis with mucus infiltration of the frontal and maxillary sinuses. Thoracic CT scan showed multiple centrilobular micronodules bilaterally, predominantly in the upper lobes, 7 mm in maximum diameter. Magnetic resonance imaging (MRI) of the lower body showed mild muscular atrophy with diffuse hypersignal in STIR sequence. Electrophysiologic studies demonstrated preserved motor and sensory conductions in all four limbs. Extensive needle electromyography revealed increased insertional activity, abundant fibrillations with positive sharp waves, and no fasciculations. A clear myopathic recruitment pattern was also found in proximal and distal muscles.

**Figure 1:**
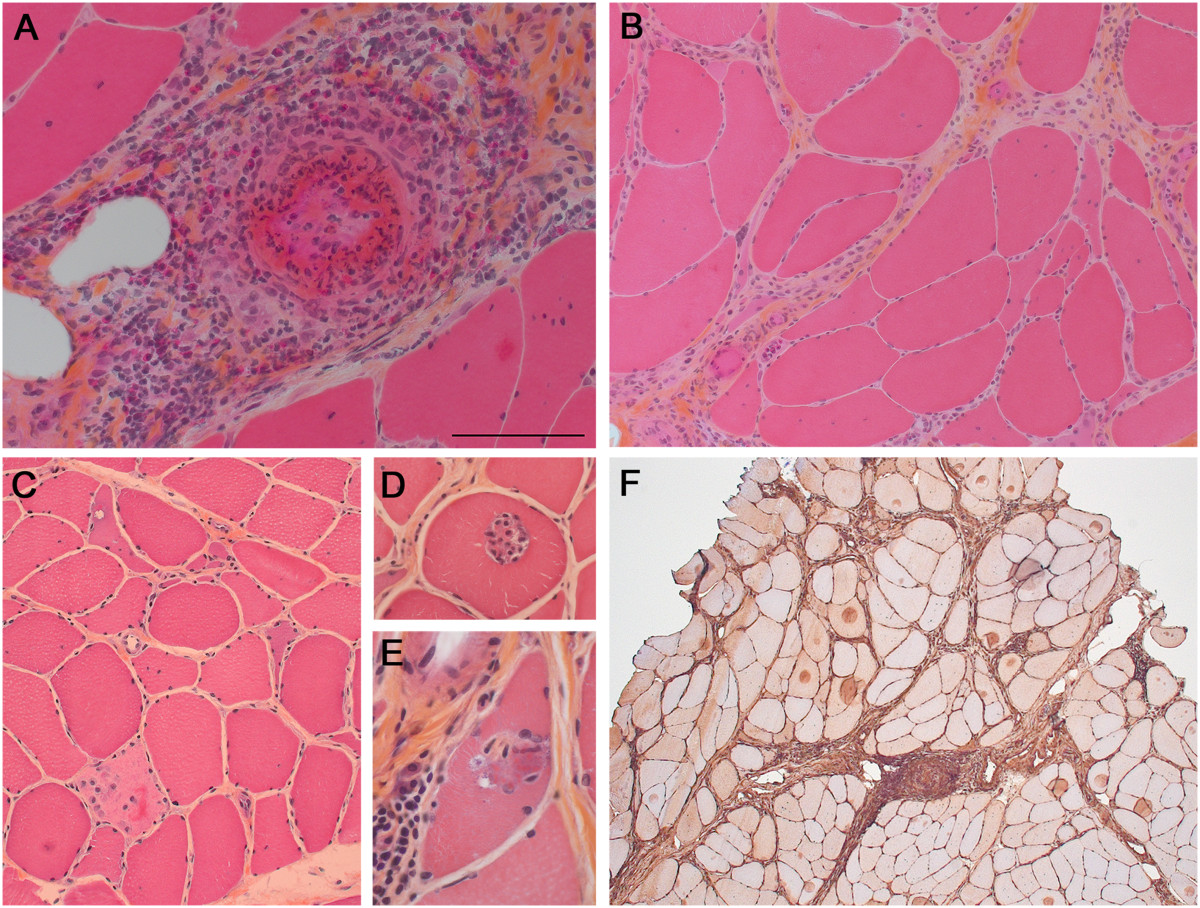
**Histopathologic evidence of vasculitis, neurogenic atrophy, and myositis.** **A-E** (HES stain), muscle cryostat sections showing: **A**, necrotizing vasculitis of medium-sized artery with abundant eosinophils; **B**, atrophic angulated fibers, nuclear clumps, and split fibers; **C**, isolated necrotic fibers; **D**, phagocytosis of non-necrotic fiber; and **E**, fiber with rimmed vacuole. **F** *,* MHC-1 immunohistochemical stain showing variable sarcolemmal and sarcoplasmic expression. *Bar* (in *A*, mm): *A*, 0.1; *B*, 0.2; *C*, 0.18; *D*, 0.12; *E*, 0.07; *F*, 0.4.

Muscle biopsy from the left biceps brachialis (which showed intermediate involvement clinically) was performed before treatment with steroids and revealed eosinophil-rich necrotizing vasculitis in perimysial blood vessels (Figure 1A)*.* There were no granulomas or endomysial eosinophilic infiltrates. Most prominent was the presence of atrophic angulated or split fibers, internalized nuclei, nuclear clumps, grouped atrophy, and foci of endomysial fibrosis (Figure 1B). Some fascicles showed isolated necrotic fibers (Figure 1C), phagocytosis of non-necrotic fibers (Figure 1D), or fibers with rimmed vacuoles (Figure 1E), but no perifascicular atrophy was seen and little inflammation was present outside areas of vasculitis. By immunohistochemistry, MHC-1 was overexpressed in muscle fibers, including in fascicles away from foci of vasculitis (Figure 1 F). Membrane attack complex (C5b9) was negative in endomysial capillaries (*not shown*). The lymphocytic infiltrate showed CD3:CD20 and CD4:CD8 ratios close to 1 (*not shown*). Apart from the vasculitis, the muscle biopsy was interpreted as consistent with chronic partial denervation with histologic and immunohistochemical evidence of nonspecific inflammatory myopathy.

After a few days in the hospital, fever subsided spontaneously and myalgia improved slightly with rest. Prednisone 80 mg and azathioprine 50 mg daily were introduced at discharge with tapering of 10 mg every 14 days. Within 2 weeks, laboratory data showed: white blood cell count, 17.0 ×10^9^/L; eosinophil count, 0.11 ×10^9^/L; creatine kinase (CK), 63 U/L; C-reactive protein, 6.9 mg/L and alanine transaminase, 32 U/L. Within 6 weeks of therapy, the patient was nearly symptom-free. Motor and sensory conductions were still unaffected and needle examination was normal with no spontaneous activity. Unfortunately, the patient suffered a relapse in weakness, which was diffuse and severe. This was attributed to the very rapid tapering of corticosteroids. Repeat EMG was identical and showed no neuropathy.

## Conclusion

Because of its heterogeneous and multisystemic presentation with potential pulmonary, cutaneous, and/or neurological involvement, EGPA can become a diagnostic challenge with a wide differential. Here, myositis and eosinophilia raised the possibility of parasitic or bacterial infections, and viral syndromes. The addition of recent travels, consumption of bear meat, and minor stool changes made parasitic infections such as trichinosis a particularly interesting possibility. Other diagnoses such as hypereosinophilia syndromes, intoxication, eosinophilia-myalgia syndrome, idiopathic eosinophilic myositis, sarcoidosis, or other connective tissue diseases were also considered. All were excluded based on the clinical information, laboratory data, and histological findings. The particularity of this case was the prominence of myalgia and weakness as presenting symptoms and the relative paucity of clinical clues suggesting EGPA.

In our patient, the diagnosis of EGPA was made based on the criteria for eosinophilia, paranasal sinus abnormality, biopsy-proven eosinophilic necrotizing vasculitis, and probable late onset asthma suggested by the recent progressive episodes of wheezing with partial response to bronchodilators and CS, as well as partial obstruction reversibility on spirometry.

Clinical findings suggestive of myositis in patients with a non-EGPA systemic vasculitis may be misleading and actually represent muscle vasculitis alone rather than true myositis. For example, a rheumatoid arthritis (RA) patient with suspected myositis based on hypersignal on MRI STIR sequence only had vasculitis and edema on muscle biopsy and no evidence of myositis [14]. In a retrospective series including 17 systemic vasculitis patients with muscle biopsy, 9 showed at least probable vasculitis but only 3 showed necrotic fibers, suggesting a low rate of myositis [15].

Our case is unusual in that to our knowledge it is the first to report histologic features of primary inflammatory myopathy, such as phagocytosis of non-necrotic fibers and sarcolemmal overexpression of MHC-1, in a patient with EGPA presenting with myalgia and weakness. These findings raise the possibility that muscle involvement in our patient may not be merely the consequence of ischemia caused by middle-sized artery vasculitis in muscle and nerve but may actually involve auto-immunity against muscle fibers. Co-occurrence of systemic vasculitis and true myositis is known to occur in overlap syndromes and mixed connective tissue diseases which generally involve a combination of lupus, scleroderma, Sjögren syndrome, rheumatoid arthritis and polymyositis, but these are not commonly associated with EGPA. In most multisystemic auto-immune diseases, specific organ involvement can vary significantly for reasons difficult to establish. Thus, there may not be a characterizable pathophysiologic trigger for the occurrence of true myositis in EGPA.

Another interesting aspect of our case is the surprisingly rapid and significant response to therapy, similar to other reported EGPA patients presenting with myalgia with or without myositis [10, 12]. In our patient, despite histological changes typical of denervation, there was no clinical evidence of neuropathy, perhaps because a pathophysiologic threshold had not yet been reached. Therefore, EGPA presenting with myalgia and weakness may be an opportunity for early diagnosis which may allow effective therapy before the onset of clinically significant vasculitic neuropathy. To assure consistency in evaluating further cases, we recommend that making the diagnosis of EGPA with myositis should require biopsy with histologic and/or immunohistochemical evidence of muscle fiber auto-immunity.

### Consent

Written informed consent was obtained from the patient for publication of this Case report and any accompanying images. A copy of the written consent is available for review by the Editor of this journal.

## Authors’ original submitted files for images

Below are the links to the authors’ original submitted files for images. Authors’ original file for figure 1

## Abbreviations

EGPA: Eosinophilic granulomatosis with polyangiitis
MHC-1: Major histocompatibility complex 1
ANCA: Antineutrophil cytoplasmic antibody
CS: Corticosteroids
CK: Creatine kinase
ANA: Antinuclear antibody
HBV: Hepatitis B Virus
HCV: Hepatitis C virus
CMV: Cytomegalovirus
EBV: Epstein-barr virus
PPD: Purified protein derivative
CT: Computed tomography
EMG: Electromyography
HES: Hematoxylin-Eosin-Saffron.
