# Supplementary figures and images for: Eosinophilic granulomatosis with polyangiitis (Churg-Strauss syndrome) presenting as diffuse myositis
Source: BMC Musculoskelet Disord. 2014 Nov 21;15:388. doi: 10.1186/1471-2474-15-388 (PMC4247662; doi:10.1186/1471-2474-15-388)

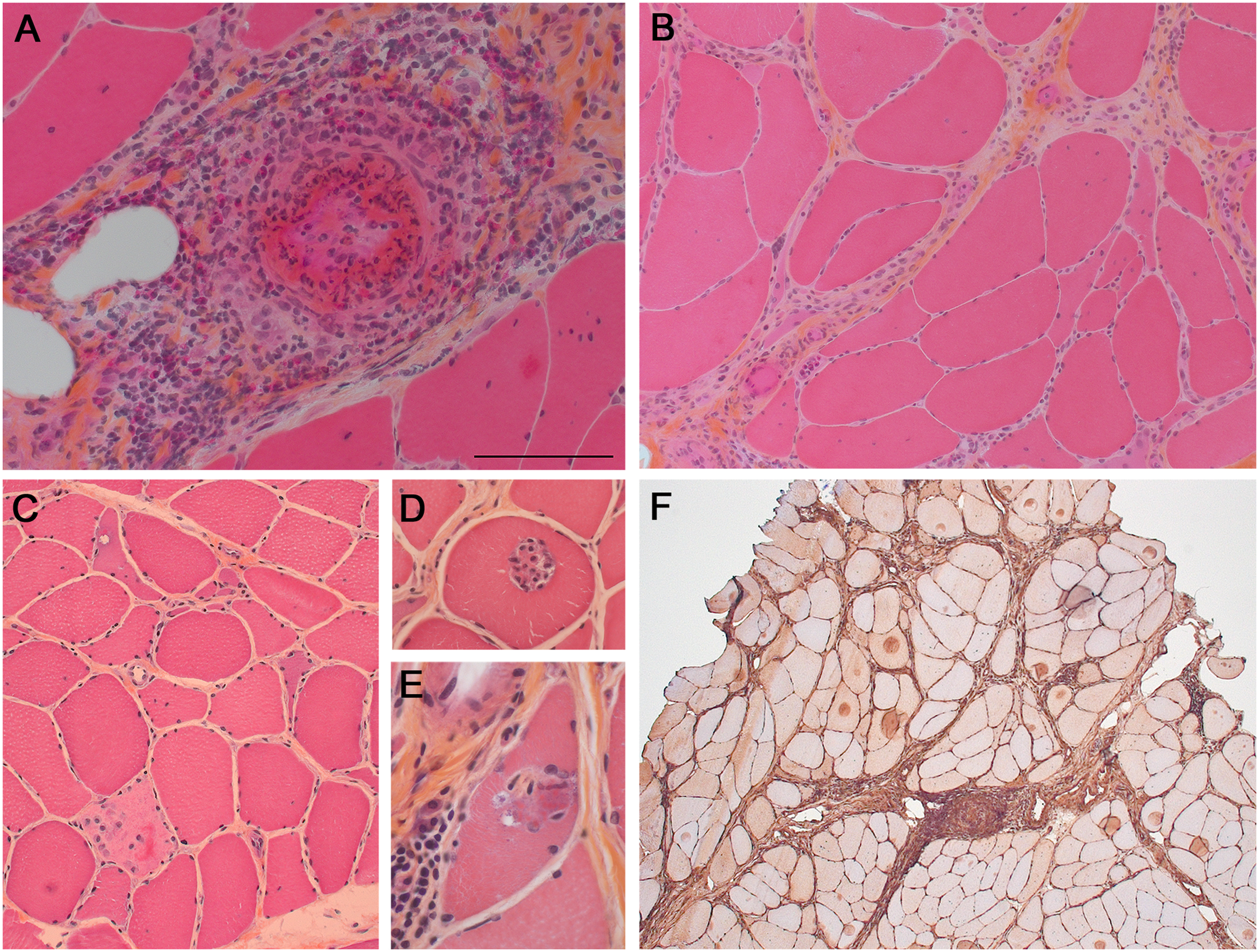

Supplement: Supplementary file 1 — Authors’ original file for figure 1 [file 12891_2014_2327_MOESM1_ESM.tif]
